# Numerical Simulation of the Mechanical Behavior of a Weft-Knitted Carbon Fiber Composite under Tensile Loading

**DOI:** 10.3390/polym14030451

**Published:** 2022-01-23

**Authors:** Mohammad Ravandi, Amirreza Moradi, Sean Ahlquist, Mihaela Banu

**Affiliations:** 1 Department of Mechanical Engineering, K. N. Toosi University of Technology, Tehran 19919-43344, Iran; armoradi99@email.kntu.ac.ir; 2 Taubman School of Architecture, University of Michigan, Ann Arbor, MI 48109, USA; ahlquist@umich.edu; 3 Department of Mechanical Engineering, University of Michigan, Ann Arbor, MI 48109, USA; mbanu@umich.edu

**Keywords:** weft-knitted composite, carbon fibers, mechanical properties, representative volume element, finite element analysis, cohesive zone model

## Abstract

Knitted textiles are a popular reinforcement in polymer composites for their high drape properties and superior impact energy absorption, making them suitable for specific composite components. Nevertheless, limited attention has been paid to modeling the mechanical behavior of knitted fabric composites since knitted textiles generally offer lower stiffness and strength. This study presents a 3D finite element (FE) modeling of a precise geometrical model of weft-knitted carbon fiber thermoplastic composite to better understand its nonlinear mechanical behavior and interface damage mechanisms under tension. Toward this end, a representative volume element (RVE) of the weft-knitted fabric composite with periodic boundary conditions (PBCs) is generated based on actual dimensions. The validity of the textile RVE to represent the macroscopic behavior was evaluated prior to analyzing the composite. The effect of fiber tow/matrix debonding during tension on the mechanical behavior of the composite is investigated using the cohesive zone model (CZM). Finally, the predicted results of the mechanical behavior of the composite with and without considering the interface failure are compared with the experimental measurements. It is found that the fiber tow/matrix interfacial strength has a significant effect on the tensile performance of the knitted fabric composites, particularly when they are subjected to a large strain. According to the simulation results, the highest tensile performance of the composite is achieved when the interfacial debonding is prevented. However, considering the fiber/matrix debonding in the modeling is essential to achieve a good agreement with the experimental results. In addition, it is concluded that stretching the fabric before composite manufacturing can substantially increase the tensile stiffness of the knitted composite.

## 1. Introduction

Structures with complex geometry, such as self-deployable systems in space applications, require specific properties, including high thermal and impact resistance as well as flexibility. It is already well known that woven/unwoven carbon fiber textiles used for reinforcing the polymers offer promising mechanical properties. Despite this fact, they are not flexible enough, mostly because of the reinforcements’ two-dimensional (2D) structure, which leads to high mechanical property performance only in the direction of the fibers. As an alternative to these reinforcements, knitted fabrics offer a capability for large 3-dimensional (3D) deformation [1,2,3]. Knitted fabrics have the potential to be employed as reinforcement in polymer composites and can be produced by robotic textile manufacturing processes, which can introduce a new generation of functional material systems with tailored structural characteristics [4,5].

Knitted fabric refers to a class of textiles formed by inter-looping of yarns through particular needle motions. The knitting process results in a high degree of yarn curvature in the fabric architecture compared to other textile manufacturing processes [6,7,8]. This high yarn curvature is the ground for the low mechanical properties of the knitted fabrics compared to non-looped textiles, in which the loads are almost uniformly distributed along the yarns. Moreover, these fabrics have a highly porous texture, which results in a low fiber volume fraction when used as reinforcement [9]. However, knitted textiles have unique advantages such as high drape properties, conformability, and high energy absorption characteristics, making them an excellent reinforcement for composites with complex geometries [5,6]. Also, knitted textiles are employed in many practical applications, such as porous materials [10].

The knitting process is traditionally divided into two general methods; warp knitting and weft knitting. Both methods are available to produce preforms using high-performance yarns, such as glass and carbon fibers for composite structures. To date, knitted composites have been utilized to manufacture several aircraft components such as wing panels, T-shape connectors, jet engine vanes, and I-beams [6,11,12].

Due to the lower mechanical properties compared to the other textile reinforcements, knitted fabric composites have not been used frequently in structural applications. Thus, few studies have been devoted to capturing the nonlinear mechanical and failure behavior of knitted fabric composites and predicting their strength. As one of the first studies, Rudd et al. [13] developed a simple model based on the rule of mixtures to predict the stiffness of knitted glass fiber composites. The comparison of the predicted stiffness with the experimental data indicated that the model needs to be modified to incorporate the fabric relaxation. In another study, Ramakrishna et al. [14] proposed an analytical model for predicting the in-plane mechanical properties of the knitted fabric laminates. Using a 2D geometrical model of the knitted fabric, their model incorporated the effects of reinforcing yarns into the rule-of-mixture while neglecting the effects of out-of-plane yarns. This assumption led to a reasonable prediction of the elastic modulus. However, there was a significant discrepancy between the composites’ predicted and experimental tensile strength.

A micromechanical approach using a 3D geometrical model of a plain weft-knitted fabric was proposed by Shekarchizadeh et al. [15] to derive the mechanical properties of the composite. They modeled a simple unit cell of a plain knitted fabric based on the geometrical model suggested by Vassiliadis et al. [16], then conducted a finite element (FE) simulation to extract the elastic properties of the composite. However, to improve the accuracy of the virtual tensile test to predict the performance of knitted composites, more precise geometrical models of knitted fabrics are required. To capture the force-determined geometry and residual stresses imposed during the knitting process, Duhovic et al. [8] performed a dynamic finite element simulation of the manufacturing process. Then, the resultant geometry was employed in a static FE analysis of the knitted fabric composite to predict the mechanical performance.

So far, many geometrical modeling strategies have been proposed to obtain the geometry of different types of textiles, such as 2D and 3D woven, braided, and knitted [5,9,17,18,19,20,21]. These geometries have been commonly employed in micro-scale and multi-scale models to estimate the elastic properties and predict the fracture behavior of the composites reinforced by these textiles. Among others, polynomial splines are more appropriate for representing the knitted loop curve [21,22]. A 3D FEA of the weft-knitted fabric was conducted by Liu et al. [22] to study the influences of the material architecture on both in- and out-of-plane deformations. However, due to the complexity of the geometric architecture of knitted fabrics, assessing the effects of these deformations on the yarn/matrix interactions in the knitted composites, and consequently, their overall mechanical behavior is still lacking.

The mechanical behavior and strength of a composite not only depend on the properties of its constituent (i.e., fiber and matrix) but also the properties of the interfaces. In general, fiber/matrix interface failure is one of the main failure modes in composites that can deteriorate their load-carrying capacity. Unlike the other types of textile composites, the influence of fiber/matrix (inter-yarn) and yarn/matrix (intra-yarn) failures in knitted composites is not well investigated in the literature.

This study aims to numerically investigate the mechanical behavior of the weft-knitted carbon fiber/Polypropylene (PP) composite through a high-fidelity simulation based on 3D FE analysis. A simulation framework is proposed to capture the large-strain nonlinear material response of weft-knitted composites. Having a proper numerical model to predict the mechanical behavior of weft-knitted composites can provide valuable insight into how damage propagation changes with fabric architecture and help engineers use the knitted fabric composites in engineering designs for particular applications.

## 2. Simulation Strategy

### 2.1. Geometrical Modelling

**Figure 1:**
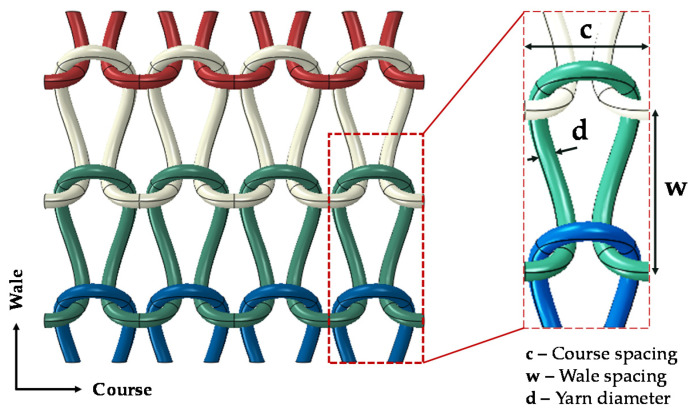
Knitting pattern of the plain weft-knitted fabric and its geometrical parameters.

Knitted fabrics are generally categorized into two main classifications: weft- and warp-knitted fabrics. Weft-knitted fabrics, which are typically produced by interlacing one yarn, are the most common and cost-effective knitted fabrics. The plain weft-knitted fabric, known as “single jersey”, was used in this study. Figure 1 illustrates the knitting pattern and the main geometrical parameters of plain weft-knitted fabric. The horizontal and vertical row of loops in knitted fabrics are known as course and wale, respectively.

**Figure 2:**
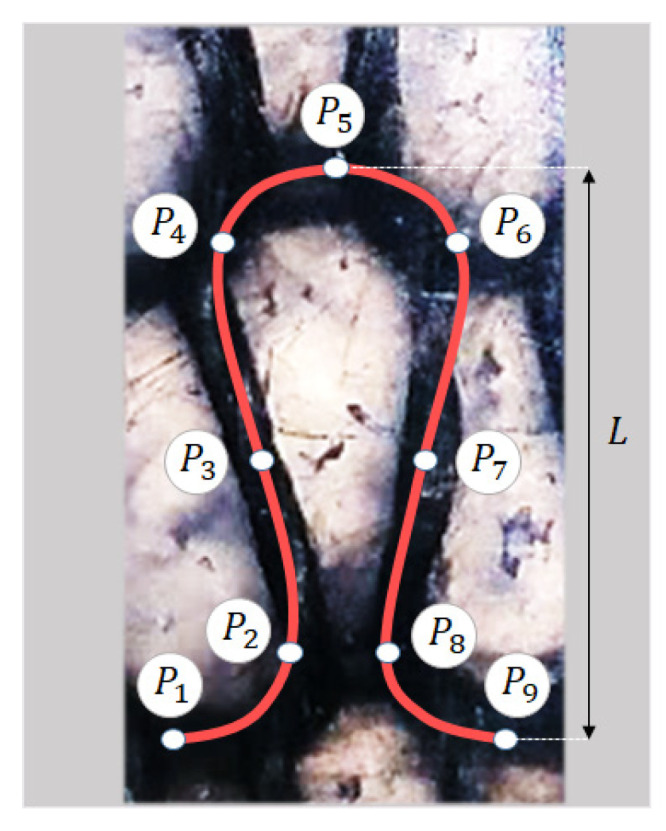
Schematic illustration of the fiber tow centerline curve of a loop of the manufactured knitted carbon fiber/PP composite, and the corresponding points ($P_{1}–P_{9}$) used to define the B-spline curve.

The 3D geometry of the plain knitted loop was created by sweeping the circular cross-section of the yarn along the spatial curve, defining the centerline path of the yarn. The yarn centerline of a periodic knitted loop was defined using a B-spline curve passing through a set of 9 points of $\left\{P_{1},\;\ldots ,\;P_{9}\right\}$, as shown in Figure 2 [21]. Due to the periodic structure, the centerline curve was defined so that the first and second derivatives remain continuous along the connected loops. The x, y, z coordinates of the points $P_{1}$ to $P_{9}$ can be calculated based on the actual geometrical parameters of the knitted fabric, i.e., course (row) spacing and wale (column) spacing of loops and yarn diameter. The x, y, and z coordinates of these points in terms of the fabric parameters are as follows:

$$\begin{array}{c} P_{1}=\left(0,0,\frac{d}{2}\right),P_{2}=\left(\frac{c+2d}{4},\frac{L-w}{2},0\right),P_{3}=\left(\frac{c}{4},\frac{L}{2},-\frac{d}{2}\right),P_{4}=\left(\frac{c-2d}{4},\frac{L+w}{2},0\right),P_{5}=\left(\frac{c}{2},L,\frac{d}{2}\right),\\ P_{6}=\left(\frac{3c+2d}{4},\frac{L+w}{2},0\right),P_{7}=\left(\frac{3c}{4},\frac{L}{2},-\frac{d}{2}\right),P_{8}=\left(\frac{3c-2d}{4},\frac{L-w}{2},0\right),P_{9}=\left(c,0,\frac{d}{2}\right) \end{array}$$

where c, d, and w were defined in Figure 1, and L is the height of the knitted loop, as shown in Figure 2. This loop is replicated in the course directions to generate a knitted row. Then, they are assembled in the wale direction with a row spacing of w to produce the complete model of the knitted fabric. The values of the geometrical parameters used in this study were obtained from the experimental specimen shown in Figure 2.

### 2.2. Periodic Boundary Conditions

**Figure 3:**
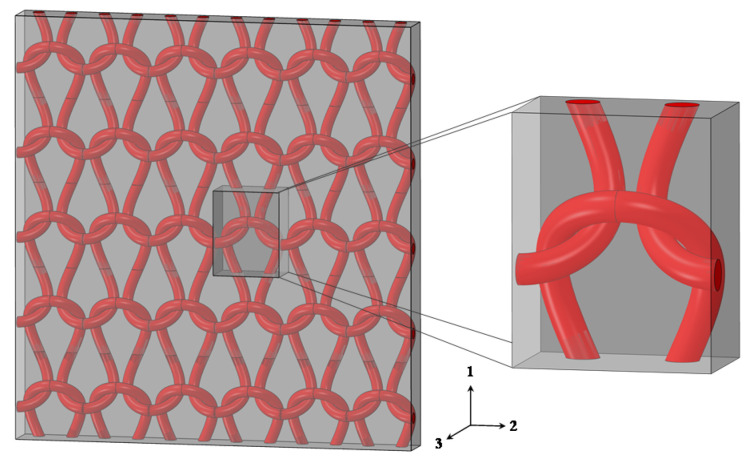
The single-layer weft-knitted composite and its RVE.

The FE analysis of a periodic representative volume element (RVE) is a valuable technique to study the material properties and estimate the effective properties of composites. In order to ensure that the assembly of RVEs always satisfies the compatibility conditions, periodic boundary conditions (PBCs) are applied. The PBCs guarantee the continuity of the displacement and traction field for the adjacent RVEs. The continuity condition of the traction can be automatically satisfied using the periodic displacement boundary conditions in the displacement-based analysis [23]. The periodic displacement boundary conditions were imposed using the linear constraint of Equation (1) between each pair of corresponding nodes on the opposite surfaces of the RVE:

(1) $$u_{i}^{j+}-u_{i}^{j-}={\bar{\varepsilon }}_{ik}\left(X_{k}^{j+}-X_{k}^{j-}\right),\;\;\;\;i=x,y,z$$

where u and X indicate the displacement and coordinate of nodes, respectively, “j+” and “j−” denote the jth pair of opposite nodes on RVE boundaries, ${\bar{\varepsilon }}_{ik}$ is the global strain of the RVE, and i=x,y,z. An Abaqus plugin was used to apply the PBCs on the RVE. The generated single-layer knitted fabric composite and the selected RVE are illustrated in Figure 3.

### 2.3. Material Model

A mesoscale 3D finite element (FE) model of the RVE of the weft-knitted fabric composite was generated and analyzed using the commercial software Abaqus/Standard. The geometrical model of the fabric was created with the assumption of a circular cross-sectional fiber tow using the approach described in the previous section. The pure matrix block of the composite was constructed by cutting out the geometry of the knitted fiber tow from a rectangular block. The fiber tow and the pure matrix were modeled using a 10-node quadratic tetrahedron element (C3D10). The complete RVE model of the knitted composite and its constituting components are shown in Figure 3. The fiber volume fraction ($V_{f}$) of the RVE—the ratio of fiber tow volume to RVE volume—is calculated to be 10.5%.

The fiber tow was assumed to be fully impregnated with the matrix, resembling a unidirectional composite with an axis of symmetry along the fiber direction and uniformity in the transverse direction. Therefore, the impregnated fiber tow was modeled as a transversely isotropic elastic material with respect to local coordinates. Due to the anisotropy and curvature, the element local coordinate system of the fiber tow was defined such that the first direction of each element coincides with the fiber direction. The material properties of the impregnated carbon fiber tow were obtained from the microscale analysis outlined in the next section.

The pure matrix was modeled as an isotropic homogeneous material with elastic-plastic behavior. The Johnson-Cook plasticity model was chosen to model the strain hardening behavior of the matrix. In this study, the effect of strain rate on the mechanical behavior was neglected due to the assumption of quasi-static loading. The static yield stress of the Johnson-Cook plasticity model is expressed as:

(2) $$\sigma^{0}=\left[A+B{\bar{\varepsilon }}^{pl}\right]\left[1-{\bar{\theta }}^{m}\right]$$

where ${\bar{\varepsilon }}^{pl}$ is the equivalent plastic strain, *A* is the initial yield strength, *B* and *n* represent the flow stress on strain hardening, and m represents the thermal softening effect. These parameters are measured at the reference temperature $\theta_{0}$, and $\bar{\theta }$ is the non-dimensional temperature defined as:

(3) $$\bar{\theta }=\{\begin{array}{cc} 0 & \theta <\theta_{0}\\ \left(\theta -\theta_{0}\right)/\left(\theta_{m}-\theta_{0}\right) & \;\;\;\;\;\;\;\;\;\;\theta_{0}\leq \theta \leq \theta_{m}\\ 1 & \theta >\theta_{m} \end{array}$$

where θ is the current temperature and $\theta_{m}$ is the melting temperature. The second bracket of Equation (2) characterizes the thermal softening of the materials when the analysis is performed at an elevated temperature (i.e., $\theta >\theta_{0})$. Since the analysis was assumed to perform at the same temperature as the material properties of the matrix obtained (i.e., room temperature), the effect of the thermal softening is eliminated from the model. Thus, the only material constants left to determine are A, B, and n. The Mcalibration software was employed to calibrate the model parameters based on a tensile test stress-strain curve of polypropylene. The Johnson-Cook plasticity model was used in conjunction with a linear elastic model to capture the elastic-plastic behavior. The material constants of the polypropylene matrix are given in Table 1.

#### Microscale Analysis of Fiber Tow

**Figure 4:**
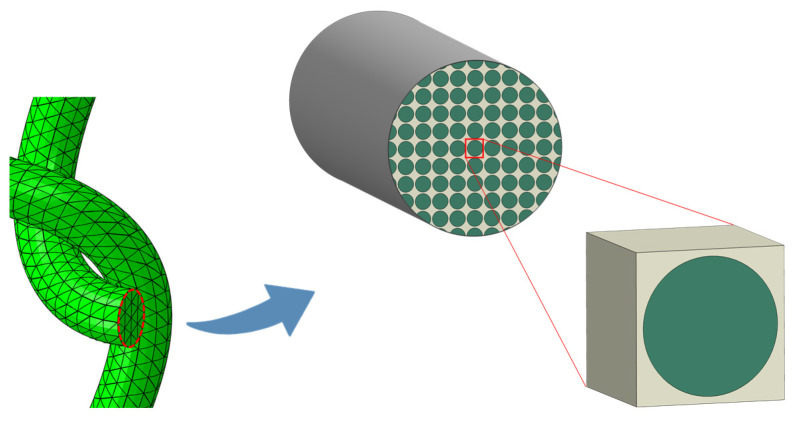
The 3D square representative UC of the impregnated fiber tow.

The effective material properties of the impregnated fiber tow were determined by conducting a micro-scale analysis, assuming that the impregnated fiber tow is a unidirectional composite with a square fiber arrangement. Thus, the 3D square representative unit cell (UC) of carbon fiber/PP composite shown in Figure 4 was constructed for the micro-scale FE analysis. The fiber volume fraction of the UC was set to 65%, which was the same as the impregnated fiber tow. In this model, the carbon fiber was treated as transversely isotropic material, while the matrix was considered as isotropic material with linear elastic behavior. The properties of the polypropylene matrix and the carbon fiber used as model inputs are given in Table 1 and Table 2, respectively.

The generated UC was analyzed with six different loading cases, where there was only one nonzero stress component in each case, to predict the material behavior of the fiber tow. The UC was meshed with 41,150 linear hexahedral elements (C3D8R). The element size was obtained after a mesh convergence analysis. The displacement-based PBC described in the previous section was adopted to define the boundary conditions for the UC. Then, the average stress and strain of the UC were calculated to obtain the effective material properties of the impregnated fiber tow, assuming that material properties of a heterogeneous material can be represented by the effective properties of an equivalent homogenous one [25].

### 2.4. Fiber Tow/Matrix Interface

The fiber tow/matrix debonding in knitted fabric composites is a complex failure mode that occurs outside the fiber tow at the interface with the surrounding matrix. In order to study the effect of the fiber tow/matrix bonding on the overall behavior of the composite, different algorithms were used to model their interface, including tie constraint, cohesive zone model (CZM), hard contact with and without surface friction.

The CZM with a bilinear traction-separation law was used to model the fiber tow/matrix interface failure when the composite is stretched. The Abaqus surface-to-surface contact algorithm with a cohesive behavior was used to define the interaction. The interface failure onset was modeled using the quadratic failure criterion [26,27]. The interface damage evolution was specified based on the fracture energy criterion, and the mixed-mode fracture energy was determined by the Benzeggagh and Kenane (B-K) relation [28]. Once the interface fails, the stresses are transmitted by means of a hard contact algorithm with a tangential friction coefficient of 0.1. Also, the hard contact algorithm with the same coefficient of friction was used for the interaction between fiber tows. The properties used in the CZM are given in Table 3.

## 3. Experimental Details

A composite panel of weft-knitted fabric and thermoplastic (polypropylene) resin was prepared to validate the numerical model. A single ply of the weft-knitted carbon fiber fabric supplied by University of Michigan Taubman School of Architecture, used as fiber preform. A polypropylene sheet with a thickness of 1.5 mm with a melting temperature of 190 °C was used as the polymer matrix. The composite panel was manufactured via the hot-pressing technique.

The knitted fabric was fixed to an aluminum mold at all four sides. Two polypropylene sheets were cut down to the size of the mold window and placed in the mold on the top and bottom of the fabric. After putting the mold lid over the cavity, the mold was placed in a hydraulic press under a pressure of 12 bar. Then, the mold was heated to 195 ℃ and held at this temperature for 15 min for consolidation. Finally, the heater was off, and the mold was left to cool down to room temperature under the same pressure. The test specimens with dimensions of 250 mm × 20 mm were cut from the cured composite panel. The final thickness of the panel was 2.16 mm, and its average fiber volume fraction was measured to be 10 ± 0.5%.

**Figure 5:**
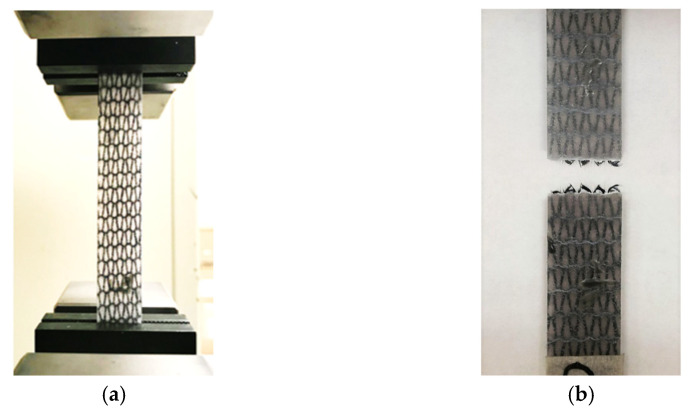
(**a**) The weft-knitted carbon fiber/polypropylene composite specimen during the tensile test and (**b**) a tested specimen.

The tensile tests were carried out in the wale direction (W) on an Instron 3345 universal testing machine with a 5 kN load cell at a cross-head displacement rate of 2 mm/min per ASTM D3039. Figure 5 shows the tensile test setup and a specimen after testing.

## 4. Results and Discussion

### 4.1. Verification of the Fabric RVE and Mesh Convergence Analysis

**Figure 6:**
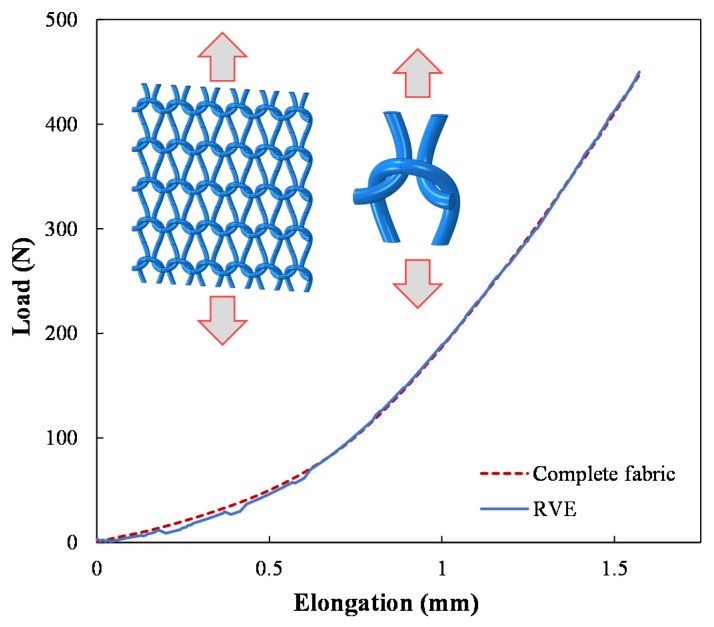
Comparison of the load-elongation curve of the complete fabric model and its RVE under tensile loading in the wale direction.

The generated RVE of the weft-knitted fabric was validated against the complete model of the fabric by comparing their mechanical response under tensile loading. Figure 6 shows the predicted tensile force against the elongation in the wale direction for both models. As can be seen, the predicted response by the RVE is in good agreement with the result of the complete model. This result assures that the RVE mode of the knitted fabric can capture the fabric’s mechanical behavior, and therefore, it can be used to generate an RVE of the knitted fabric composite. Besides, it is evident from Figure 6 that the weft-knitted fabric has hyperelastic behavior. This behavior is attributed to the particular geometry of the knitted fabric, which allows the fiber tows to move easily and have large deformations. As a result, more fibers get aligned with the loading direction as the fabric stretches further.

**Figure 7:**
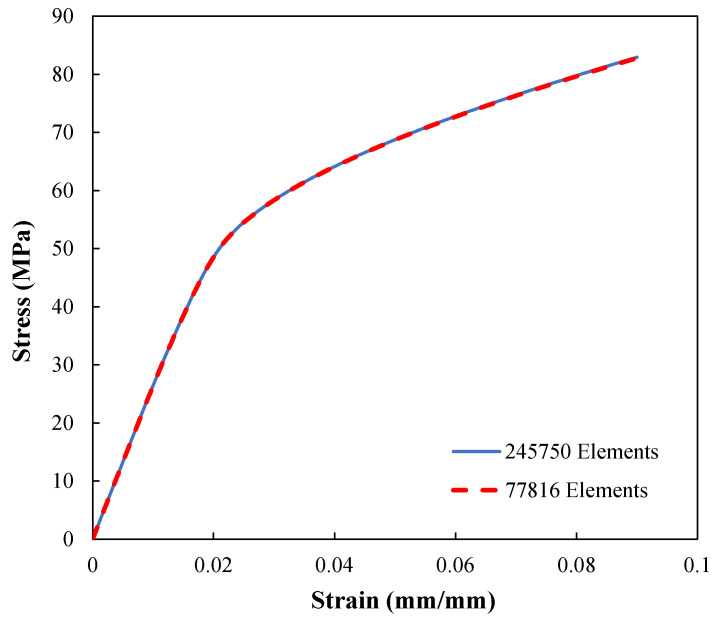
Mesh refinement study of the composite RVE.

In addition, a mesh refinement study is conducted with two different mesh densities to ensure the mesh-independency of the composite RVE analysis results. Figure 7 indicates that the model is independent of the mesh resolution.

### 4.2. Effect of Fiber Tow/Matrix Interface

**Figure 8:**
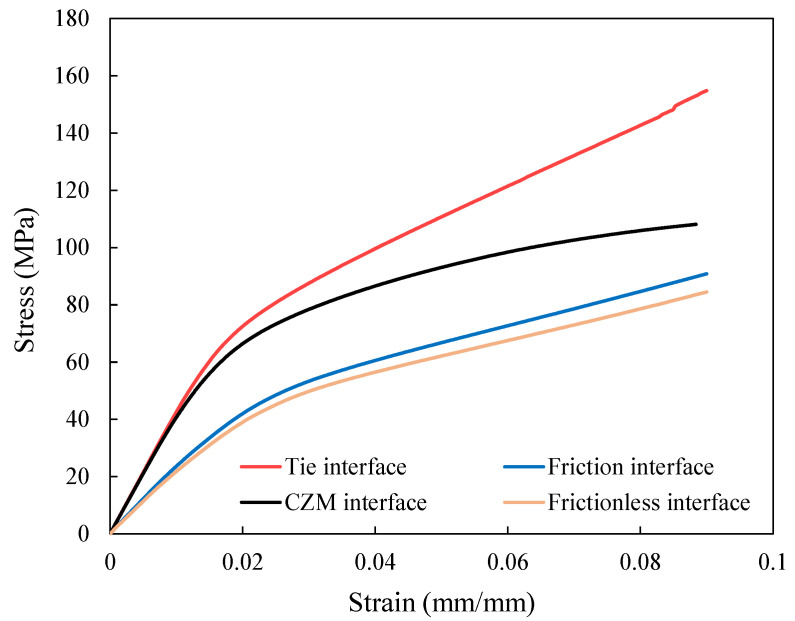
Prediction of stress-strain response of the knitted composite in the wale direction using different methods to model the fiber tow/matrix interfaces.

The mechanical response of the weft-knitted fabric composite has been analyzed as a function of the fiber tow/matrix interface. Figure 8 shows the influence of the fiber tow/matrix interface on the mechanical behavior of the composite loaded in the wale direction using four different types of interfaces. In the tie interface, there is no debonding since fiber tows and matrix surfaces are constrained to each other. However, failure can occur at the fiber tow/matrix interface during loading in the case of the cohesive interface. The two other studies, frictional and frictionless Interfaces, are designed to evaluate the effects of considering friction between fiber tow and matrix surfaces after debonding on the mechanical response of the RVE. In the frictional interface, the interaction between the fiber tow and matrix is modeled as hard contact with frictional tangential behavior, whereas friction is neglected in the frictionless interface.

It is evident from Figure 8 that the bonding between fiber tow and matrix has a significant effect on the mechanical behavior and load-carrying capacity of the knitted composite, especially when it is subjected to large deformation. The tie interface offers the highest mechanical behavior as a result of the perfect bonding at the interface. On the other hand, the frictionless interface has the lowest one as there is no interaction between fiber tow and matrix surfaces except hard contact.

The RVE with CZM interface behaves approximately like the tie interface at small strains since the cohesive failure has not been initiated yet. However, with increasing deformation, its behavior diverges from the tie interface due to interfacial damage growth. These results reveal that the fiber tow/matrix debonding has a remarkable impact on the load-carrying capacity of the composite. Consequently, the CZM properties can have a significant effect on the composite behavior by tuning the interfacial strength. The CZM with lower fracture toughness diverges further from the tie interface model because the interfacial failure occurs at a relatively early stage of loading (i.e., small strains), offering lower load-carrying capacity.

In addition, Figure 8 shows that the frictional interface can slightly enhance the mechanical response compared to the frictionless model. After interfacial failure, friction is the only interaction between debonded surfaces, preventing them from sliding freely. As a result, considering frictional behavior in the interface is a more realistic assumption.

**Figure 9:**
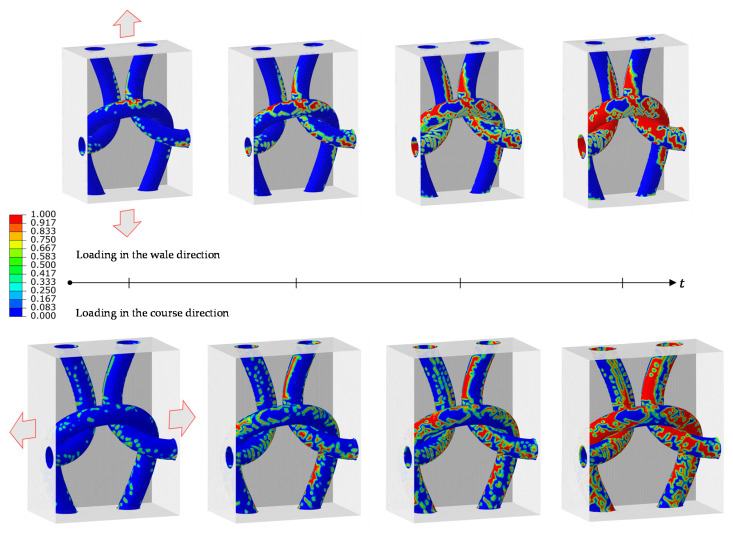
Cohesive damage evolution at the fiber tow/matrix interfaces of the knitted composite RVE during tension in the principal directions.

Figure 9 illustrates the cohesive damage evolution on the fiber tow’s surfaces of the knitted composite during tension along the wale and course directions, at four different time steps. It can be observed that for the loading in the wale direction, the cohesive damage forms and propagates on the horizontal part of the fiber tows—perpendicular to the loading direction, till debonding occurs all over the fiber tows. In contrast, the formation of the cohesive damage is on the vertical surfaces of the fiber tows when loaded in the course direction. It is evident from this figure that the cohesive damage, i.e., fiber tow/matrix debonding, is an extensive failure mode in the knitted composite. This behavior is mainly due to the complexity of the knitted fabric structure, which enables the fiber tow to move and rotate extensively during tension or compression.

### 4.3. Comparison of Simulation and Experimental Results

**Figure 10:**
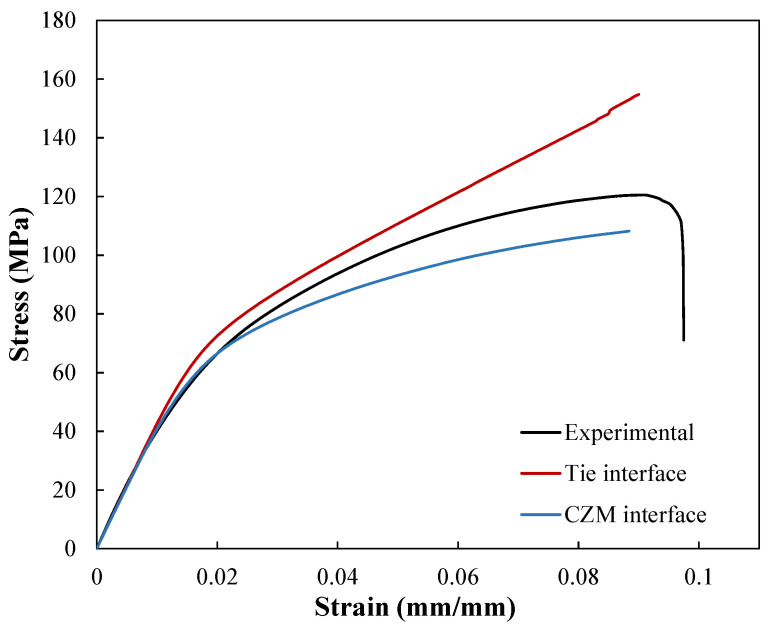
Stress-strain curves of the numerical simulation versus the experimental tensile test.

The engineering stress-strain curves obtained from the simulation of the RVE with tie and CZM interfaces are compared with the experimental tensile test result in Figure 10. As can be seen, up to 1% strain, both models can accurately predict the linear elastic response of the composite. However, there are some discrepancies between the simulation results and experimental measurement. The tie interface model starts to overestimate the mechanical response as deformation increases. This error is attributed to the model’s inability to capture the failure at the fiber tow/matrix interfaces.

The model with the CZM interface, on the other hand, appears to provide a better fit to the actual behavior for a wider range of strain since the debonding at the fiber tow/matrix interface during loading is captured by this model. Despite this, the model’s prediction for strains above 2.2% is slightly below the actual response. This discrepancy between the simulation and experimental results can be mainly attributed to the simplifying assumptions made in modeling the knitted composite. The current model makes a number of assumptions, including representing the fiber tow by a constant radius circular cross-section, the absence of fiber bridging in the fiber tow/matrix debonding, considering complete impregnation of the fiber tow by the polymer resin, and neglecting the residual stresses caused by stretching the fabric before manufacturing the composite. To the authors’ knowledge, the residual stresses do not significantly influence the mechanical response of the composite. However, the constant circular cross-section assumption for modeling the fiber tow and neglecting the effect of the fiber bridging can alter the mechanical behavior by affecting the fiber tow/matrix interface failure. In reality, there are some broken fibers protruding from the surface of the fiber tow. These broken fibers are created during the knitting process due to the brittleness of carbon fibers. However, these fibers lead to improvement in interfacial bonding by bridging the crack.

**Figure 11:**
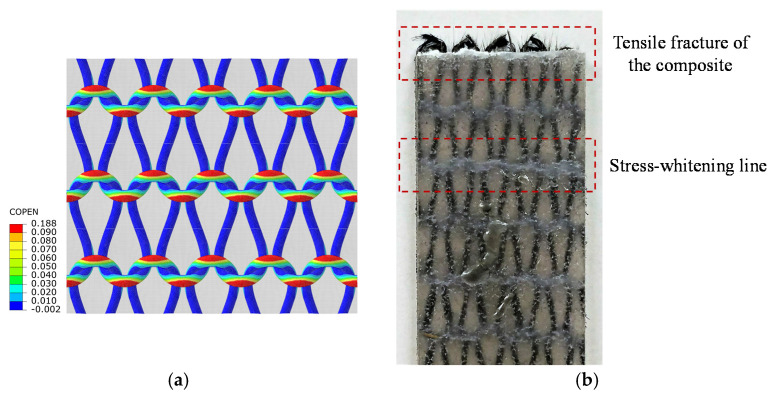
(**a**) The COPEN index shows the separation of fiber tow/matrix interface after loading in the wale direction; (**b**) The fiber tow/matrix separations (stress whitening) which occurred in the test specimen due to the tensile loading in the wale direction.

Figure 11a depicts the separation of the fiber tow from the surrounding matrix in the knitted composite loaded in the wale direction based on the simulation results of the RVE. The figure also shows a tested specimen of the knitted composite under the same conditions for comparison. According to the simulation results discussed in the previous section, interface failure is dominant around the knitted loops. This corresponds with the stress whitening marks visible on the tested specimen (Figure 11b). The stress whitening lines, which formed along the knitted loop rows perpendicular to the load direction, indicate the formation and accumulation of microcracks and reorientation of polymer chains. It can also be observed from Figure 11b that the fracture of the specimen occurred at the stress-whitening region.

Therefore, based on the numerical results and experimental observations, it can be concluded that the areas near the loops are the critical region in the knitted composite and the fiber tow/matrix interface failure is one of the major failure modes there.

### 4.4. Mechanical Response of Weft-Knitted Carbon Fiber/PP Composite

**Figure 12:**
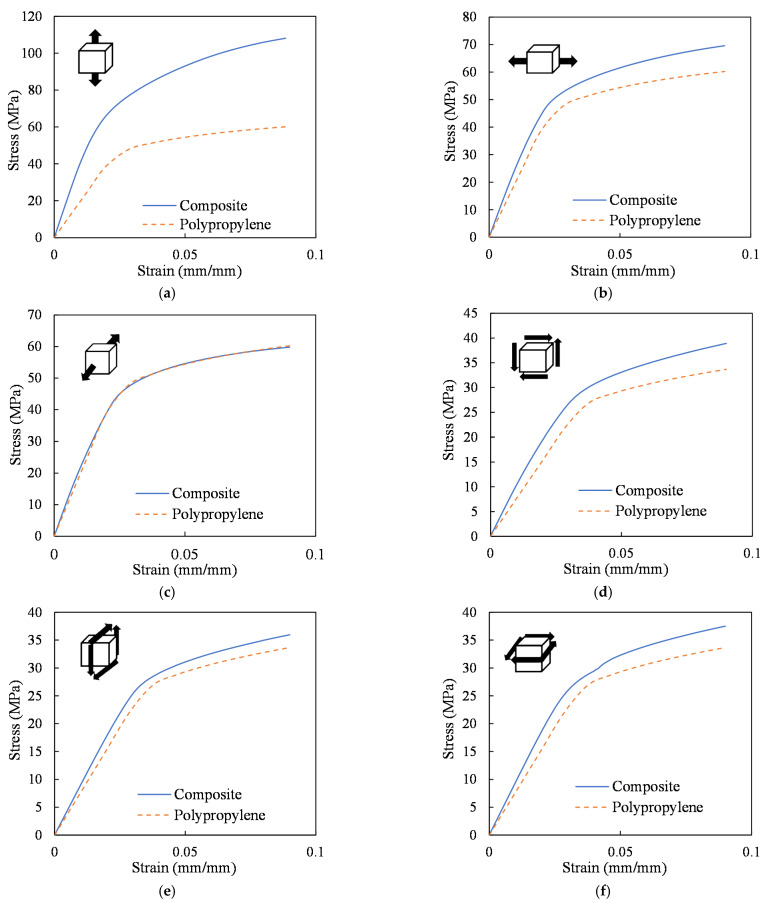
Stress-strain curve of the composite RVE under six periodic loading conditions compared with the pure PP; (**a**) $\sigma_{11}-\varepsilon_{11}$ (wale direction); (**b**) $\sigma_{22}-\varepsilon_{22}$ (course direction); (**c**) $\sigma_{33}-\varepsilon_{33}$ (thickness direction); (**d**) $\sigma_{12}-\varepsilon_{12}$; (**e**) $\sigma_{13}-\varepsilon_{13}$; (**f**) $\sigma_{23}-\varepsilon_{23}$.

The elastoplastic material behavior of the weft-knitted composite obtained from the simulation of the RVE by applying six different periodic displacement loading conditions (three cases of normal loads and three cases of shear loads) are shown in Figure 12. Here, simulations were carried out using the CZM at the fiber tow/matrix interface, and the effect of interface failure was considered. The results are also compared with pure matrix response in order to evaluate the performance of the knitted fabric reinforced composite with a $V_{f}$ of 10.5%. According to the results of Figure 12, the wale direction yields higher tensile properties compared to the course direction.

As can be seen, the mechanical behavior of the knitted composite can be divided into two main regions: the linear elastic response and the nonlinear plastic region. The initial elastic modulus along the wale direction is 65% higher than the course direction and 88.6% higher than the modulus in the thickness direction. The higher mechanical behavior in the wale direction is associated with the larger wale spacing in this knitted fabric. This particular texture is due to the fabric’s stretching before processing the composite, which leads to more aligned fibers in the wale direction. The mechanical behavior of RVE in the thickness direction is mainly dependent on the matrix properties since there are very few reinforcements along this direction.

The lower mechanical behavior of the composite in the course direction is associated with the fabric’s hyperelastic behavior. With no pre-applied stretch, the stiffness of the reinforcing fabric in the course direction is minimal. Therefore, its additive effect on the overall stiffness of the composite is insignificant. Consequently, similar to the thickness direction, the mechanical behavior of the composite in the course direction would be dominated by the matrix. Hence, stretching the knitted fabric can significantly affect the mechanical properties of its composite.

The consequence of stretching fabric can be divided into two main parts: creating prestress and changing the fabric geometry. The latter plays a vital role in altering the mechanical behavior of the composite. As shown in Section 4.1, the knitted fabric has a hyperelastic material response, and its stiffness increases significantly during stretching. The impact of geometry has been considered in the simulation by generating the fabric’s geometry model using an actual composite specimen in which the fabric was stretched in the wale direction before impregnation with polypropylene (Figure 2). The effect of fiber tow prestress was neglected as it had a minor impact on the results.

**Figure 13:**
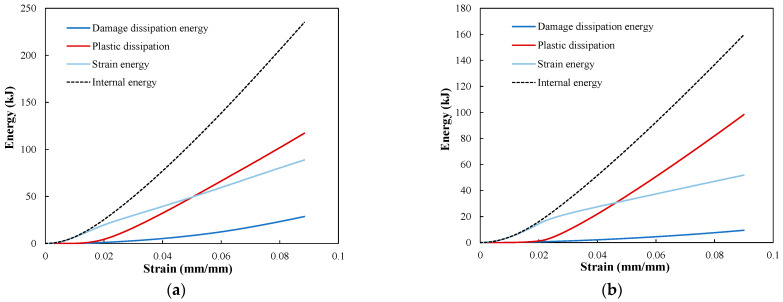
Comparison of energy dissipated by cohesive damage and plasticity of the matrix with elastic strain energy for (**a**) loading in the wale direction and (**b**) loading in the course direction.

The energy dissipated by the fiber tow/matrix interface failure and matrix plasticity are compared to the elastic strain energy in Figure 13 as a function of strain. The cohesive damage, plasticity, and elastic strain energy are the main energy absorption mechanisms as their summation is almost equal to the total internal energy of the RVE. Figure 13 indicates that the energy dissipated by matrix plasticity during tension is significantly higher than the energy dissipated by the cohesive damage. The energy dissipated by cohesive damage is 12.2% of the total internal energy of the RVE loaded in the wale direction, whereas the contribution of matrix plasticity is almost 49.9%. The rest of the internal energy is a recoverable energy corresponding to the elastic strain energy. The considerable plastic dissipation energy is related to the low fiber volume fraction ($V_{f}=10.5\%$) and the complexity of the knitted fabric structure, which leads to complex deformation of the matrix. Consequently, the energy absorbed by the matrix, composed of elastic energy and plastic dissipation, is the primary energy absorption mechanism during deformation.

## 5. Conclusions

In this study, a 3D finite element simulation framework to study the tensile behavior and interfacial damage mechanisms of the weft-knitted carbon fiber thermoplastic composite was presented. The procedure of creating a precise geometrical model of the knitted fabric composite was provided. A representative volume element (RVE) of the knitted composite was used for finite element analysis by applying periodic boundary conditions. The interaction between fiber tow surfaces and surrounded matrix was molded using four different algorithms, namely cohesive interaction, tie constraint, frictional and frictionless interface, in order to assess the effects of fiber tow/matrix debonding and friction on the tensile behavior of the composite. Finally, the simulation results were validated by an experimental study.

The results showed that the weft-knitted fabric reinforcement could improve the elastic modulus of the polymer by at least 118.4% and 32.3% in the wale and course direction, respectively, at a fiber volume fraction as low as 10%. According to the simulation results, stretching the knitted fabric prior to composite processing could significantly improve the knitted composite mechanical behavior due to the fabric’s hyper-elastic behavior.

The simulation results revealed that the fiber tow/matrix interaction significantly impacts the load-carrying capacity of the knitted fabric composite, particularly at large deformations. Comparing the results of the four different fiber tow/matrix interaction algorithms with the experimental results showed that the CZM led to a more accurate prediction of the mechanical behavior of the composite due to its ability to capture the fiber tow/matrix debonding. Due to the fact that the knitted composites are often recommended for applications involving large deformations, it is essential to consider the effects of fiber tow/matrix interfacial failure in the FE simulation of knitted composites to achieve a more realistic prediction of their mechanical behavior.

The numerical framework proposed in this paper provides a valuable tool to design a superior knitted composite for particular applications. Based on the required performance for the composite, the textile manufacturing parameters, such as wale spacing, course spacing, and yarn diameter, can be determined through conducting a parametric study. Another potential area for future investigation is incorporating a good multiscale yarn damage model into the current simulation to predict the composite’s ultimate strength.

## Figures and Tables

**Table 1:** Elastic and plastic material constants of polypropylene [24].

Young Modulus, E (GPa)	Poisson Ratio, v	A (MPa)	B (MPa)	*n*
2002.03	0.3	43	94	0.5

**Table 2:** Elastic properties of carbon fiber used in the microscale analysis.

$E_{11}$ (GPa)	$E_{22}\;=\;E_{33}$ (GPa)	$G_{12}\;=\;G_{13}$ (GPa)	$G_{23}$ (GPa)	$v_{12}\;=\;v_{13}$	$v_{23}$
250	22.4	22.1	8.3	0.3	0.35

**Table 3:** Cohesive zone properties used in the modeling of fiber tow/matrix interface.

$G_{IC}$ (KJ/m^2^)	$G_{IIC}\;=\;G_{IIIC}$ (KJ/m^2^)	$\sigma_{I}^{C}$ (MPa)	$\sigma_{II}^{C}\;=\;\sigma_{III}^{C}$ (MPa)	η
4	8	35	60	1

## Data Availability

Not applicable.
